# Micro and Macro Element Composition of *Kalanchoe integra* Leaves: An Adjuvant Treatment for Hypertension in Ghana

**DOI:** 10.1155/2015/579497

**Published:** 2015-10-01

**Authors:** S. Frimpong-Manso, I. J. Asiedu-Gyekye, J. P. Naadu, G. T. Magnus-Aryitey, A. K. Nyarko, D. Boamah, M. Awan

**Affiliations:** ^1^Department of Pharmaceutical Chemistry, UGSOP, Legon, Ghana; ^2^Department of Pharmacology and Toxicology, UGSOP, Legon, Ghana; ^3^Geological Survey Department, Accra, Ghana

## Abstract

Two samples, water extract and blended whole leaves, of fresh *Kalanchoe integra* leaves (Crassulaceae), a traditional antihypertensive medicine used in Ghana, were analyzed with Energy Dispersive X-Ray Fluorescence spectroscopy (EDXRF). Analysis revealed 12 macro and 26 micro elements in both extracts. Further quantitative assessment of the results for amounts of elements that are pharmacologically significant revealed that the amounts of calcium, potassium, and magnesium present in the extracts could be correlated to its traditional usage in managing hypertension and arrhythmias. However, heavy metals (lead and inorganic arsenic) detected in the extracts may pose a threat at doses normally used traditionally for the treatment of hypertension.

## 1. Introduction

Since time immemorial, man has used herbs and potions as medicines, but it is only around the mid-nineteenth century that serious efforts were made to isolate and purify active principles from these remedies [1]. Despite the availability of well-developed synthetic organic compounds, there are high patronage plant-based medicines used either as nutritional supplements or raw materials for pharmaceutical industry, and increasingly for general healthcare delivery resulting from its perceived efficacy and low cost [2]. Indeed, in some countries, efficacious plant preparations are recognized by the national health programmes [3].

Like every other drug or medicine, the activity of plant-based medicines is due to their pharmacologically active compounds [4], which include alkaloids, glycosides, flavonoids, sugars, tannins, and many other organic constituents. There is the probability that these secondary metabolites may interact or interfere with the micro and macro elements present in plants. These interactions and interferences may damage or enhance the bioavailability of micro and macro elements within plant tissues [5]. The presence of toxic heavy metals in medicinal plants can on the other hand pose a threat to the health of consumers [6]. Thus for safety of consumers, the World Health Organization states maximum permissible levels in raw plant materials for only cadmium (0.3 mg kg^−1^), arsenic (1 mg kg^−1^), and lead (10 mg kg^−1^) [7]. The geochemical properties of the soil, aerial, and/or aquatic environment as well as the capacity of plants to accumulate elements selectively from their surroundings determine the contents of elements in plants.* Kalanchoe pinnata* (*K. pinnata*), a medicinal plant used in Ghana, has broad pharmacological properties that include immunosuppression, antimicrobial activity; wound healing, antinociceptive, anti-inflammatory, antioxidant, analgesic and anticonvulsant, antidiabetic, and antihypertensive activities among others [8, 9].

Phytochemical analysis of* Kalanchoe* species revealed the rich content of flavonoid and phenolic compounds that have antioxidant activity [10]. Though the species also contains bioinorganic elements such as magnesium, calcium, potassium, sodium, iron, and zinc [11] correlation between pharmacological activity of the plant and these macro and micro elements has not been established. This present study seeks to investigate the macro- and microelemental content of* Kalanchoe integra* and correlate them with its traditional usage in the management of hypertension in Ghana.

## 2. Method

Leaves of* Kalanchoe integra*, collected from the Legon Botanical gardens and authenticated at the Herbarium of the Botany Department of the University of Ghana, were washed and spread in an aerated room at room temperature overnight.

The plant samples were treated by two methods. With the first method (sample A), 3.5 kg of* K. integra* leaves and 5 liters deionized water (25°C) were blended with a heavy duty blender. The watery paste was spread in an aerated room to dry at room temperature overnight and the resultant cake homogenized with the heavy duty blender and sieved (number 180; mesh size 300 *μ*m) [6] to obtain the required particle size of the sample.

In the second method (sample B), mimicking the traditional method of extraction, 2.8 kg of* K. integra* was blended with 1 liter/kilogram hot deionized water (90°C and 95°C). The mixture was covered and left to stand overnight in a water bath maintained at 60°C. The watery portion (infusion) was filtered off using 0.45 *μ*m millipore cellulose ester filters, the infusion freeze-dried and the residue homogenized as in sample A.

Energy Dispersive X-Ray Fluorescence spectroscopic (EDXRF) analysis (Spectro X-Lab 2000 spectrometer), fast, nondestructive and unit cost of elemental measurement relatively economical, at Geological Survey Department, Accra, Ghana, was used to simultaneously measure the elements in the* Kalanchoe* samples. The method employs three-axial geometry, thereby reducing background noise by radiation polarization. The monochromatic radiation emitted from the X-ray tube on a secondary target is applied to excite the atoms of the sample.

The Spectro X-Lab 2000 spectrometer used was equipped with Rh anode and 400 W Pd X-ray tube, a 0.5 mm Be end window tube, a Si (Li) detector (resolution of 148 eV – 1000 cps Mn K*α*), available targets (Al_2_O_3_ and B_4_C used as a BARKLA polarizer), an HOPG (High Oriented Pyrolitic Graphite) as a BRAGG polarizer, Al, Mo, and Co as secondary target, and a 0.5 mm Be side window. It has a carousel (circular rotating sample changer) inside a sample chamber with a capacity of 20 sample holder disc (32 mm) for sequential sample analyses. The radiation chamber was cooled using liquid nitrogen. Its computer-based multichannel analyzer, SPECTRO X-Lab Pro Software package (Turboquant), controlled and computed spectral analysis, collecting and storing data as well as evaluating data. Combination of these different targets gave a typical detection limit for light elements (Si, Al, Mg, and Na) in the range of 25–50 ppm. For heavy metals 1–5 ppm was the limits of detection. The spectrometer was factory calibrated applying a number of international rock standards.

The samples were kept in an oven at 60°C prior to pelletation. Due to their morphology and the loose nature, triplicate weighed samples, 4 g/sample, were added separately to 0.9 g Fluxana H Elektronic BM-0002-1 (Licowax C micropowder PM-Hoechstwax) as binder; the mixture was homogenized using the RETSCH Mixer Mill (MM301) for 3 minutes and pressed manually with SPECAC hydraulic press for 2 minutes with a maximum pressure limit of 15 tons (15000 kg) into pellets of 32 mm in diameter and 3 mm thickness for subsequent XRF measurements. Time between pelleting and measurement was kept short to avoid deformation of the flat surfaces of the pellets [6, 12, 13].

The results presented as mean, standard deviation (±) (SD), coefficient of variation (CV), and *P* values were used to analyse the results (Table 1).

## 3. Results

Triplicate four grams of each sample analyzed with EDXRF gave thirty-eight (38) elements with twelve (12) major {sodium (Na), magnesium (Mg), aluminum (Al), silicon (Si), phosphorus (P), sulphur (S), chlorine (Cl), potassium (K), calcium (Ca), titanium (Ti), manganese (Mn), and iron (Fe)} and twenty-six (26) minor {vanadium (V), chromium (Cr), cobalt (Co), nickel (Ni), copper (Cu), zinc (Zn), gallium (Ga), arsenic (As), rubidium (Rb), strontium (Sr), yttrium (Y), zirconium (Zr), niobium (Nb), molybdenum (Mo), antimony (Sb), iodine (I), cesium (Cs), barium (Ba), lanthanum (La), cerium (Ce), hafnium (Hf), tantalum (Ta), lead (Pb), bismuth (Bi), thorium (Th), and uranium (U)} elements in percentage weight per 100 grams weight of sample (%w/w) and parts per million (ppm). Fourteen (14) elements (Table 1) including Na, Mg, K, and Ca (believed to be involved in the pathophysiology of hypertension and dysrhythmias) [14, 15] and seven (7) important heavy metals (Ni, Fe, Cu, Zn, Mn, Pb, and Cd) were selected for quantification and evaluation. Elements of interest either in %w/w or ppm were simply converted to their respective amounts in milligrams. For example an average of triplicate measurements of elements such as potassium (K) in percentage was converted as (4.78 + 4.98 + 4.50 = 14.26/3 = 4.75/100*∗*4000 mg = 190 mg 4 g^−1^) and arsenic (As) in parts per million (ppm) was calculated as an average of triplicate measurement 0.4/1000000*∗*4000 = 0.0016 mg 4 g^−1^. Their measured quantities (mg 4 g^−1^) ranged from 190.09 (K) to less than 0.0016 (As, Y) for sample A and 35.61 (Ca) to less than 0.002 (Ga, Th) for sample B. It could be observed from Table 1 that the elements implicated with the physiology and etymology of hypertension, K (190.09; 34.98), Ca (107.52; 37.60), Cl (19.14; 4.29), and Mg (13.23; 4.39), for samples A and B, respectively, were very prominent. Sample A contained significantly higher levels of Ni, Zn, and Mn (0.012; 0.047; and 0.108, resp.) while sample B contained more of Fe and Cu (1.308 and 0.539, resp.) with *P* values less than 0.05. No significant difference was observed in the quantities of Pb (3.33 and 3.20) in both samples A and B, respectively.

Based on work done by Walpole et al. [14], 2012, a dose of 100 mg/kg for an average adult body weight of 60.70 kg was used to calculate mean quantities with respect to the tolerable upper intake levels (UL). The percentages of minerals contained in 6.070 mg of each sample with respect to tolerable upper intake levels (UL) are as shown in Table 2. Except K {values reflecting adequate intake (AI)}, all other values were calculated according to the UL.

## 4. Discussion

Completely randomized analysis of variance (ANOVA) was used for statistical evaluation. *P* values less than 0.05 were considered significant.

The aqueous extract (mimicking the traditional method of preparation in Ghana) and powdered whole leaf samples contained relatively higher amounts of Na, Ca, K, and Mg. These are known to play a key role in hypertension management [16, 17]. They also contained heavy metals (Mn, Fe, Ni, Mo, V, Cu, and Zn); their obtained values were well above tolerable upper intake level as shown in Table 2.

The aqueous extract, however, contained relatively lower amounts of the heavy metals. It should be noted that the concentrations of macro elements in infusions are controlled by their content in the initial plant material. The amount of magnesium in both samples (A = 5734.71 and B = 1905.40) expressed in percentage compared to UL were relatively high for a dose of 100 mg/kg for an average adult body weight of 60.7 kg. This supports its pathophysiological usage to treat hypertension [18].

Potassium in samples A and B (2307.92 and 12543.03, resp.) expressed in percentage were also above UL. The vasodilatation role of potassium in treatment of hypertension [19] and its use in salt supplementation therapy in hypertensive patients [20] is well researched.

Compared to potassium and magnesium, both samples contained significantly different amounts of calcium far above the recommended UL. The possible role of calcium in treating hypertension has been well researched, but appropriateness and inappropriateness of altered salt and calcium intake remains a debate [21].

Sodium was also found to be significantly high in both samples. These observations caution users of* Kalanchoe integra* as dietary supplement for calcium [22] and sodium in hypertension treatment, public health, and regulatory bodies to monitor and regulate the usage of this plant as an antihypertensive agent to avoid complications.

The low levels of heavy metals in the extract are significant as it eliminates toxicity arising from heavy metals.

## 5. Conclusion

Between the two methods of preparation, the aqueous extraction (may be effective in reducing the potential mineral toxicity associated with injection of the leafy preparation) is recommended. EDXRF fast, nondestructive, and lower cost of measurement is best for first-hand information about the mineral levels in plant materials. The high content of calcium, potassium, and magnesium found in the sample studied with an EDXRF spectroscopy may have a possible role to play in the management of hypertension and arrhythmia among the traditional Ghanaian users of the herb. Hypertensive patients who use* Kalanchoe integra* leaves as dietary supplement for calcium and its salt, especially those sensitive to salt, require monitoring to reduce possibility of worsening their hypertensive state. However, the levels of heavy metals also present may pose a threat to invalidated dosage of the plant.

## Figures and Tables

**Table 1:** Mean (mg/4 g sample), standard deviation, coefficient of variance (CV%), and *P* values for 14 selected elements.

No.	Element	Mean and standard deviation		CV%		*P* value
		Sample A	Sample B	Sample A	Sample B	
1	Na	6.35 ± 0.61	1.91 ± 0.08	9.61	4.19	>0.001
2	Mg	13.23 ± 0.82	4.39 ± 0.83	6.20	18.92	>0.001
3	P	7.17 ± 0.48	6.97 ± 0.75	6.70	10.76	<0.089
4	K	190.09 ± 9.59	34.98 ± 4.77	5.05	13.64	>0.001
5	Ca	107.52 ± 8.34	35.60 ± 2.09	7.76	5.87	>0.001
6	Mn	0.11 ± 0.01	0.05 ± 0.01	9.09	20.00	>0.001
7	Fe	0.46 ± 0.04	1.31 ± 0.02	8.70	1.53	>0.001
8	Pb	<3.33 ± 0.02	<3.20 ± 0.00	<0.60	<0.00	<0.374
9	Ni	12.00 ± 0.69	6.93 ± 2.66	5.75	38.38	>0.033
10	Mo	<5.20 ± 0.69	<4.67 ± 1.77	<13.27	<37.90	<0.547
11	V	<15.20 ± 0.69	<10.93 ± 0.61	<4.54	<5.58	>0.004
12	Cu	30.27 ± 6.50	53.87 ± 4.64	21.47	8.61	>0.007
13	Cr	<29.60 ± 2.12	36.13 ± 12.25	<7.16	33.91	<0.414
14	Zn	461.07 ± 28.46	40.40 ± 3.56	6.17	8.81	>0.001

**Table 2:** Mean quantities of selected elements in 6,070 mg^*∗∗*^ of samples expressed as percentages of UL or AI [15, 23].

No.	Element	UL or AI (mg)	% in 6,070 mg A	% in 6,070 mg B
1	Na	400	2407.77	723.34
2	Mg	350	5734.71	1905.40
3	P	400	3948.54	3505.93
4	K^*∗*^	2300^*∗*^	12542.03	2307.92
5	Ca	2500	6526.46	2161.16
6	Mn	11	1495.43	634.59
7	Fe	45	1554.37	4412.22
8	Ni	01	1821.00	1052.08
9	Mo	02	394.55	354.11
10	V	02	1281.44	921.71
11	Cu	10	459.30	817.43
12	Zn	40	1749.17	153.27

K^*∗*^ is the adequate intake (AI), ^*∗∗*^6,070 was obtained using the preceding paragraph: based on 100 mg/kg body weight and an average body weight of 60, 70 theoretical values of 6,070 were obtained.

## References

[1] Graham L. P. (1995). *An Introduction to Medicinal Chemistry*.

[2] Elless M. P., Blaylock M. J., Huang J. W., Gussman C. D. (2000). Plants as a natural source of concentrated mineral nutritional supplements. *Food Chemistry*.

[3] Quazi M. A., Tatiya A., Khurshid M., Nazim S., Siraj S. (2011). The miracle plant (*Kalanchoe pinnata*): a phytochemical and pharmacological review. *International Journal of Research in Ayurveda and Pharmacology*.

[4] Gareth T. (2007). *Medicinal Chemistry*.

[5] Eyal R. (2007). Micro-elements in agriculture. *Practical Hydroponics and Greenhouses*.

[6] World Health Organization (1998). *Quality Control Methods for Medicinal Plant Materials*.

[7] Carvelho M. L., Ferrerira J. G., Amorim P., Marques M. L. M., Ramos M. T. (1997). Heavy metals in macrophyte algae using X-ray fluorescence. *Environmental Toxicology Chemistry*.

[8] Rácz L., Bumbálová A., Harangozó M., Tölgyessy J., Tomeček O. (2000). Determination of cesium and selenium cultivated mushrooms using radionuclide X-ray fluorescence technique. *Journal of Radioanalytical and Nuclear Chemistry*.

[9] Richardson D. H. S., Shore M., Hartree R., Richardson R. M. (1995). The use of X-ray fluorescence spectrometry for the analysis of plants, especially lichens, employed in biological monitoring. *Science of the Total Environment*.

[10] Asiedu-Gyekye I. J., Antwi D. A., Bugyei K. A., Awortwe C. (2012). Comparative study of two *Kalanchoe* species: total flavonoid, phenolic contents and antioxidant properties. *African Journal of Pure and Applied Chemistry*.

[11] Stroffekova O., Plankova A., Janosova V., Sykorova M., Havranek E. (2008). Determination of Fe, Zn, Pb, Cd and Se content in medicinal plants by X-ray fluorescence analysis and galvanostatic stripping chronopotentiometric analysis. *Acta Facultatis Pharmaceuticae Universitatis Comenianae*.

[12] Biswas S. K., Chowdhury A., Das J., Zahid Hosen S. M., Uddin R., Rahaman S. M. (2011). Literature review on pharmacological potentials of *Kalanchoe pinnata* (Crassulaceae). *African Journal of Pharmacy and Pharmacology*.

[13] Ghasi S., Egwuibe C., Achukwu P. U., Onyeanusi J. C. (2011). Assessment of the medical benefit in the folkloric use of *Bryophyllum pinnatum* leaf among the igbos of Nigeria for the treatment of hypertension. *African Journal of Pharmacy and Pharmacology*.

[14] Walpole S. C., Prieto-Merino D., Edwards P., Cleland J., Stevens G., Roberts I. (2012). The weight of nations: an estimation of adult human biomass. *BMC Public Health*.

[15] Food and Nutrition Board (1997). *Standing Committee on the Evaluation of Dietary Reference Intakes*.

[16] Nguyen H., Odelola O. A., Rangaswami J., Amanullah A. (2013). A review of nutritional factors in hypertension management. *International Journal of Hypertension*.

[17] Houston M. (2011). The role of magnesium in hypertension and cardiovascular disease. *The Journal of Clinical Hypertension*.

[18] Sontia B., Touyz R. M. (2007). Role of magnesium in hypertension. *Archives of Biochemistry and Biophysics*.

[19] Macgregor G., Markandu N. D., Smith S. J., Banks R. A., Sagnella G. A. (1982). Moderate potassium supplementation in essential hypertension. *The Lancet*.

[20] Adrogué H. J., Madias N. E. (2007). Sodium and potassium in the pathogenesis of hypertension. *The New England Journal of Medicine*.

[21] Haddy F. J., Vanhoutte P. M., Feletou M. (2006). Role of potassium in regulating blood flow and blood pressure. *The American Journal of Physiology—Regulatory Integrative and Comparative Physiology*.

[22] Resnick L. M. (1999). The role of dietary calcium in hypertension: a hierarchal overview. *American Journal of Hypertension*.

[23] Trumbo P., Yates A. A., Schlicker S., Poos M. (2001). Dietary reference intakes: vitamin A, vitamin K, arsenic, boron, chromium, copper, iodine, iron, manganese, molybdenum, nickel, silicon, vanadium, and zinc. *Journal of the American Dietetic Association*.

